# A team-based translational journal club: Understanding the translational research highway

**DOI:** 10.1017/cts.2019.414

**Published:** 2019-10-04

**Authors:** Carol Feghali-Bostwick, Jillian Harvey, Carissa Hasseler, Diana Lee-Chavarria, Perry Halushka

**Affiliations:** 1 Department of Medicine, College of Medicine, Medical University of South Carolina, Charleston, SC, USA; 2 Department of Healthcare Leadership and Management, College of Health Professions, Medical University of South Carolina, Charleston, SC, USA; 3 South Carolina Clinical and Translational Research Institute, Medical University of South Carolina, Charleston, SC, USA; 4 Departments of Pharmacology and Medicine, College of Graduate Studies, Medical University of South Carolina, Charleston, SC, USA

**Keywords:** Journal club, translational research, team science, dissemination and implementation, predoctoral trainees

## Abstract

The mission of the National Center for Advancing Translational Science (NCATS) is to catalyze the generation of innovative methods and technologies that will enhance the development, testing, and implementation of diagnostics and therapeutics across a wide range of human diseases and conditions.^1^ NCATS funded a predoctoral TL1 training grant at our institution. We developed a novel team-based Translational Journal Club utilizing three-member teams to find a basic science paper and two clinical study papers that covered a single therapeutic, either a pivotal study or a dissemination and implementation study; one member of the team presented a paper on the above topics in successive weeks. In addition, the trainees attended lectures on: how to design a pivotal clinical trial, dissemination and implementation, and drug development from a basic science discovery through its approval. From these presentations, the trainees appreciated the T0 to T3/4 continuum and its challenges. They also attended sessions on how to present scientific concepts, making them better communicators. The trainees found the Translational Journal club to be very rewarding, illuminating, and providing a much better understanding of the translational research processes required to develop new therapies.

## Introduction

Journal clubs organized by either departments or training grants are commonplace in academic medical centers. Most of these journal clubs focus on a single paper per session, and the topic is often related to the focus of the training grant, mentor, or the department and usually goes into great detail to dissect the strengths and weaknesses of the study design. The format of these journal clubs is fairly standard and explores the paper section by section with a critique and discussion of each section with an overall wrap up. While the importance of the paper may be discussed, it is usually within a very narrow focus and does not span consideration of the translation of the discovery all the way through to a therapeutic agent. Rarely is there a succession of presentations with a single theme.

The mission of the National Center for Advancing Translational Science (NCATS) is to catalyze the generation of innovative methods and technologies that will enhance the development, testing, and implementation of diagnostics and therapeutics across a wide range of human diseases and conditions.^1^ NCATS funds T32 training grants through the Clinical and Translational Science Award Program titled TL1 Clinical Research Training Awards. Our institution is the recipient of one of these grants focused on training predoctoral trainees. The grant funds 10 trainees yearly, including both PhD and MD/PhD trainees in their graduate years. Given the mission of NCATS, we decided to develop a novel Translational Journal Club that would educate TL1 trainees on the process of translation from discovery to dissemination and implementation. Since another focus of NCATS is the promotion of team science, our approach would also promulgate a team-based approach. The trainees were divided into groups of three and tasked with working as a team to choose three papers that would exemplify translational research from T0 to T3/4. Thus, the Translational Journal Club focused on the translation of fundamental discoveries and also working as a team.

## Methods

The schedule for the Translational Journal Club is outlined in Table 1. Trainees were divided into teams of three, with one team having four trainees. The teams were purposefully chosen to provide the greatest diversity of the members’ scientific backgrounds. For example, trainees came from the departments of biochemistry and molecular biology, neuroscience, regenerative medicine and cell biology, and biostatistics and epidemiology. All were in a PhD or MD/PhD training program. The teams were charged with working together and selecting the papers for presentation; the rationale for the choice of papers was solely the team/trainees’ responsibility. The three papers to be discussed were distributed to all of the trainees prior to the team’s three presentations. The program directors did not participate in the selection process but approved the selected publications, attended all the presentations, and provided comments when appropriate. In addition, the trainees’ mentors were strongly encouraged to attend their individual mentee’s presentation and provide additional feedback. There were two sessions on how to present scientific/research data to prepare them for presenting to their peers and at national meetings, including the Association for Clinical and Translational Sciences annual meeting.

**Table 1.** Schedule for the Translational Journal Club. The first five sessions were presentations given by faculty. Weekly presentations were made by a different member of the team

Sessions	Presenter
Meeting with trainees to discuss new journal club	
Presentation of a basic discovery that was implemented into practice (treatment of cystic fibrosis)	
How to: platform presentations, poster presentations, and a TED type talk	
How to: present to the lay audience	
How to: design a pivotal clinical trial	
Dissemination and implementation	
Basic research phase	Team 1
Clinical trial phase	Team 1
ACTS conference	
Dissemination and implementation phase	Team 1
Basic research phase	Team 2
Clinical trial phase	Team 2
Dissemination and implementation phase	Team 2
Basic research phase	Team 3
Clinical trial phase	Team 3
Dissemination and implementation phase	Team 3
Entrepreneurship talk	
Discussion with trainees about lessons learned	

**Fig. 1.**
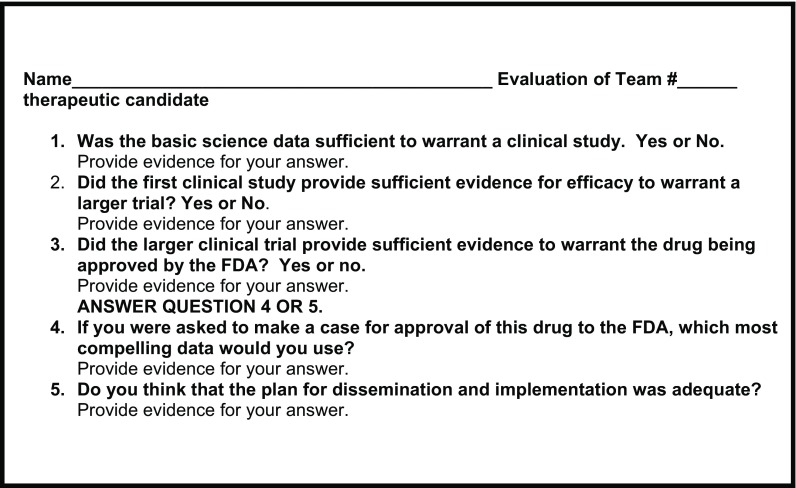
Translational Journal Club articles evaluation form. The article evaluation form was formatted using REDcap. The trainees were given the following instructions: Attached are five questions that pertain to the series of journal articles that each team presented. For each of the first three questions, there is a yes or no answer. Once you have chosen yes or no, provide 3–4 sentences backing up your choice. You will then have to answer either question 4 or 5. You will have 1 week to turn in your answers. Your final grade for the Translational Journal Club will be based on your answers.

One member of the team presented a publication in successive weeks: (1) a basic science publication that ultimately led to a clinical trial, (2) a corresponding clinical trial publication, and (3) a large-scale clinical trial publication or a publication that provided guidance on the use of the therapeutic clinically (dissemination and implementation). All trainees were encouraged to participate in discussion about the presentations. After all of the team’s presentations, the trainees were given a series of questions pertaining to the three papers and they had 1 week to answer them (Fig. 1). Their answers were graded and returned with comments; the trainees were graded honors/pass/no pass for their answers to the questions and also as their final grade for the Translational Journal Club.

To assess the impact of the Translational Journal Club and the perceived value of participation, the trainees were given an electronic survey to complete (Table 2) at the end of the semester and after they received their final grade for the Translational Journal Club. Survey questions were developed by a multidisciplinary team for the purpose of program evaluation and journal club improvement. Questions were evaluated by the team for clarity, content, and comprehensiveness related to journal club objectives. Survey questions asked the respondents their level of agreement using a Likert Scale (Strongly Disagree = 1; Disagree = 2; Neutral = 3; Agree = 4; Strongly Agree = 5). Students were also given the Net Promoter Score (NPS) survey item, which is commonly used to assess customer satisfaction^2^ by assessing the respondent’s willingness to recommend the journal club to a friend/colleague (0–10, where 0 is not at all likely and 10 is extremely likely). In addition, four open-ended items were included to collect more detailed information on: (1) How the journal club differed from the student’s prior journal club experiences?; (2) The student’s primary reason for scoring the NPS “likelihood to recommend” question; (3) The team process for selecting papers; and (4) How the journal club can be improved for next year?. The study was classified as program evaluation/quality improvement by the Institutional Review Board.

**Table 2.** Translational Journal Club Evaluation. The survey was administered confidentially. Rating Scale: Strongly Disagree = 1; Disagree = 2; Neutral = 3; Agree = 4; Strongly Agree = 5

	Mean	Range	SD
Participation in the journal club increased my understanding of team science	4.2	3–5	0.6
Participation in the journal club increased my understanding of translational science from discovery (T1) to dissemination (T4)	4.6	3–5	0.7
Participation in the journal club increased my understanding of dissemination and implementation science	4.4	3–5	0.7
The journal club facilitated interaction with students from different disciplines	4.9	4–5	0.3
Each student brought specific expertise that contributed to the journal club discussions	4.8	4–5	0.4
The journal club format promoted discussion	5	5–5	0
The review questions challenged my understanding of the research process from discovery and drug development through dissemination	3.7	2–5	1.1

## Results

Our goal was not only to enhance the trainees’ understanding of the processes from T0 to T3/4, but also to challenge them to work outside of their comfort zones. At the same time, we wanted the trainees to appreciate the power of working as a team to explore an area for which they were not familiar and learn from each other. They collaborated on topic identification and paper selection. The trainees chose the topic based on several different rationales. For example, one team chose papers associated with Rituximab because one of the members saw a patient receiving the drug in a clinic that she attended as a requirement for the TL1 program. In another situation, the papers were chosen because of interest in the disease (hepatitis C) due to familiarity with a patient with the disease. A third was chosen for the novelty of the therapeutic approach (siRNA). The trainees often supplemented their presentations with information from sources other than the publications, demonstrating that they embraced the idea of developing an in-depth understanding of both the data in the paper and additional background information. This was a significant factor for the presentations since the trainees had, at best, limited prior knowledge concerning the basic science that ultimately led to the therapeutic. Similarly, they did not have significant clinical knowledge but were able to present the clinical papers in a cogent manner.

To ensure that the trainees comprehended the concepts behind the papers, they were given a series of five questions to answer after each team’s presentation of the three papers. The trainees were able to provide cogent answers to defend their choice of a yes or no answer for each question. However, as noted in the survey (Table 2) (score 3.7), the trainees felt that the questions were not very challenging. Clearly, a more challenging set of questions will need to be developed.

A REDCap survey was administered at the end of the semester, after receipt of the final grade, to assess the trainees’ opinion of the structure and perceived value of the Translational Journal Club. The quantitative results of the survey are shown in Table 2. The questions that dealt with increasing their understanding of team science, dissemination and implementation, and translational science were all rated 4.2 or higher out of 5. Of particular relevance and rated at 5 was that the format promoted discussion.

Certain common anecdotal themes/comments emerged from the survey. When asked if they would recommend the Translational Journal Club to their colleagues on a scale from 0 to 10, with 0 being least likely and 10 being most likely, the average score was 8.7 ± 0.9 (mean ± SD). The trainees all stated that this was a very unique journal club format and they had not experienced a similar format before. Some descriptions of the Translational Journal Club were, “fun, truly enjoyed attending, looked forward to attending, engaging, entertaining, informative and provided a unique opportunity to interact with peers.” The trainees stated that they liked the continuity of the presentations and the discussions; in particular, getting the “big picture” was an important component. They also liked not dissecting the paper as is often done in the typical journal clubs, although there were critiques and discussions. They all gained more appreciation for how a basic science discovery proceeds through clinical trials (translational process) and ultimately becomes a drug, and the time that it takes to develop a new therapeutic. Interestingly, there were no negative comments. There were constructive recommendations about including a clinician’s perspective, exploring topics outside of drug discovery, and revising the final questionnaire.

In addition to the survey, the program director and associate program director met with the trainees at the end of the semester to solicit their thoughts on the Translational Journal Club. A presentation that the trainees found most interesting was the development of the therapeutics for cystic fibrosis, given by one of our clinical faculty whose expertise is in cystic fibrosis and who has participated in many clinical trials. Seeing the development of a basic science discovery into a new drug used in the clinic was most illuminating for them; in particular, they noted the length of time that it took from a fundamental discovery to an approved drug. A consensus suggestion was for a clinician with expertise in the disease area being discussed to join the discussion, so that the trainees could get a better appreciation of the disease that was being treated and the established therapeutic approaches. They also found it interesting to discover how practicing physicians get information about new drugs. A topic suggested for future journal clubs to further enhance their understanding of the T0–T3/T4 process was a lecture about the FDA approval process for new drugs.

## Discussion

This paper describes a team-based translational science approach journal club that to our knowledge is unique and previously unreported. Deenadayalan et al.^3^ described the “characteristics of successful journal clubs included regular and anticipated meetings, mandatory attendance, clear long- and short-term purpose, appropriate meeting timing and incentives, a trained journal club leader to choose papers and lead discussion, circulating papers prior to the meeting, using the internet for wider dissemination and data storage, using established critical appraisal processes and summarizing journal club findings.” Our Translational Journal Club implemented many of those characteristics. One significant difference was that we used the team approach for choosing the papers rather than a trained journal club leader. In this regard, we feel that the trainees benefited greatly from the Translational Journal Club because they had to demonstrate that the chosen papers would stand up to a critical/rigorous assessment and questions from classmates, the program directors, and/or mentors. We used a questionnaire at the end of the team presentations to assess the summarization and comprehension of the three papers.

We conducted a Pubmed search looking for descriptions of journal clubs that utilized a similar approach. We found several that had an implementation-based approach but none that emphasized a T0–T3/4 approach. One paper^4^ described a journal club that created a liaison between practicing physical therapy clinicians and academic physicians to impart an evidence-based approach to physical therapy procedures. It utilized a three-step approach of evaluating randomized clinical trials, systematic reviews, diagnostic papers, and clinical guidelines. It appeared that most of the discussions were led by the academic physicians. Another journal club focused on a multidisciplinary approach to burn therapies.^5^

Overall, the Translational Journal Club was viewed positively by the trainees. They noted gaining a greater appreciation of translational research, the challenges associated with it, and the significant length of time it takes from discovery to approval and implementation of a new therapeutic. The trainees also learned to work together as a team to choose the papers for discussion. Based on the feedback that we received from the trainees, one addition to be made in future years is to enlist participation of a clinician to be part of the team.

In summary, we have devised a novel team-based Translational Research Journal Club that can be easily implemented by other training programs or departments.
